# α_5_β_1_ integrins in hepatocytes act as receptors for bile acids with a (*nor*)ursodeoxycholane scaffold

**DOI:** 10.1186/2047-783X-19-S1-S13

**Published:** 2014-06-19

**Authors:** Michele Bonus, Annika Sommerfeld, Dieter Häussinger, Holger Gohlke

**Affiliations:** 1 Department of Mathematics and Natural Sciences, Institute for Pharmaceutical and Medicinal Chemistry, Heinrich Heine University, 40225 Düsseldorf, Germany; 2 Clinic of Gastroenterology, Hepatology and Infectious Diseases, Heinrich Heine University, 40225 Düsseldorf, Germany

## 

**Figure 1:**
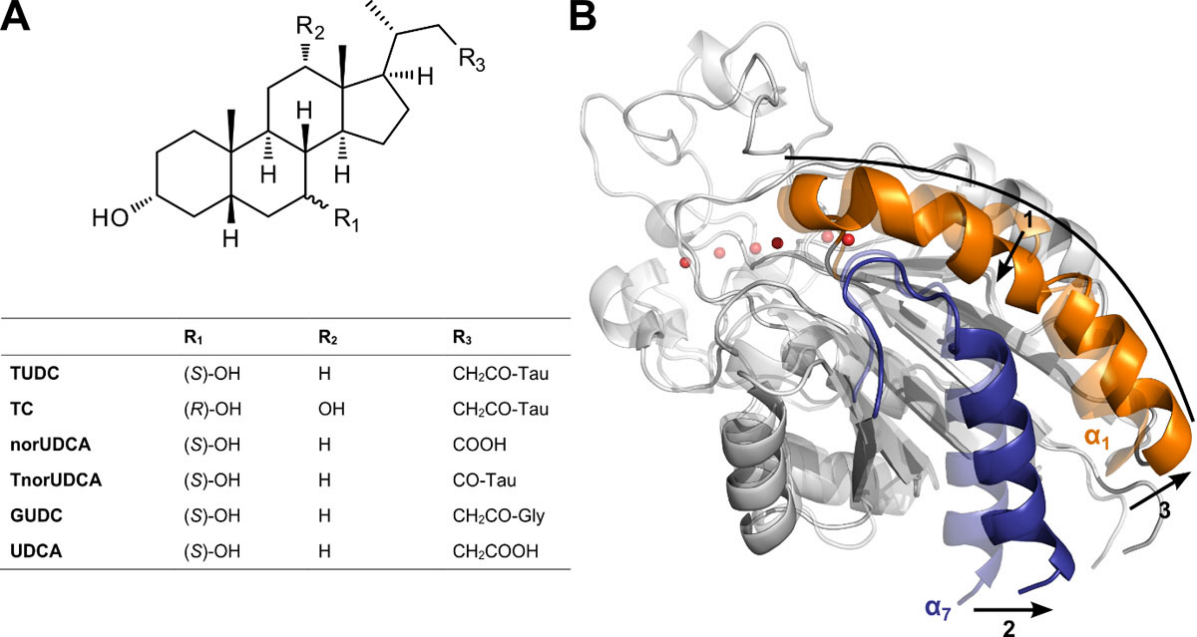
(A) Scaffold and R-groups of the investigated bile acid derivatives. (B) Overlay of the starting structure of MD simulations of α_5_β_1_-integrin bound to TUDC (transparent) and the structure closest to the average as determined over the trajectory of 200 ns length (solid). For clarity, only the βA domain of α_5_β_1_-integrin is shown together with three Mg^2+^ ions (red spheres); TUDC was omitted. Three regions of characteristic conformational changes in the βA domain are visible: I) straightening of helix α_1_ (orange); II) T-junction formation between helices α_1_ and α_7_; III) tilting of helix α_7_ (blue).

Ursodeoxycholic acid (UDCA) is a standard treatment in several cholestatic liver diseases (Figure 1A) [1]. *In vivo*, conjugation with taurine occurs rapidly and yields tauroursodeoxycholic acid (TUDC), which has been shown to promote choleresis by triggering the insertion of ATP-dependent transport proteins (e.g., the bile salt export pump (Bsep) and the multidrug resistance protein-2 (Mrp2)) into the canalicular membrane [2]. TUDC-induced recruitment of Bsep results from activation of focal adhesion kinase (FAK), phosphatidylinositol 3-kinase (PI3 kinase), and c-Src, which leads to downstream activation of extracellular signal-regulated kinases (Erks) and p38 mitogen-activated protein kinase (p38^MAPK^). Upon hepatocyte swelling, either induced by exposure to a hypoosmotic environment or insulin, α_5_β_1_ integrins become activated and trigger similar signaling events towards choleresis. α_5_β_1_ Integrins may also become activated by a swelling-independent way as previously shown by exposing hepatocytes to pathophysiological concentrations of urea [3]. Both TUDC-induced and swelling-dependent signaling were abolished in the presence of an antagonistic, RGD-motif containing hexapeptide (G*RGD*SP).

These findings led us to hypothesize that α_5_β_1_ integrin will act as a receptor for TUDC in hepatocytes. We tested this hypothesis in a combined experimental and computational study [4]. Immunofluorescence staining on cryosections of isolated perfused rat liver (IPRL) revealed the active conformation of β_1_ integrin within 1 min after addition of TUDC at a concentration of 20 µM. Furthermore, phosphorylation of Erk-1 and -2 as well as activation of the epidermal growth factor receptor were induced by TUDC within the same time span. These effects were sensitive to inhibition by G*RGD*SP but insensitive to the presence of an inactive control peptide (GRADSP). As TUDC does not affect hepatocyte volume, which excludes that TUDC triggers integrin activation osmotically, these findings demonstrated that TUDC directly activates α_5_β_1_ integrins and triggers signaling events towards choleresis. While swelling-induced β_1_ integrin activation occurs primarily in the plasma membrane, TUDC-induced β_1_ integrin activation occurs primarily in the cytosol of hepatocytes. We demonstrated that the presence of the Na^+^/taurocholate cotransporting polypeptide (Ntcp) is required for the latter. The need to uptake and/or concentrate TUDC inside the hepatocyte for β_1_ integrin activation to occur may explain why TUDC primarily acts in the liver.

In order to provide insights at a molecular level as to how TUDC activates α_5_β_1_-integrin, a complex structure of a homology model of the ectodomain of α_5_β_1_-integrin and TUDC was generated by molecular docking and subsequently subjected to molecular dynamics (MD) simulations of 200 ns length [4]. These simulations revealed pronounced conformational changes in three regions of the βA domain of the integrin (Figure 1B): I) Helix α_1_ straightens and becomes continuous; II) this leads to a tighter packing between the top of helix α_7_ and the center of α_1_, which has been characterized as “T-junction formation” in an X-ray structure of integrin α_IIb_β_3_ bound to a ligand as well as in computational studies of agonist-bound integrins; III) as a result, helix α_7_ moves downwards and outwards, which imposes a torque on the hybrid domain. The induced rotational motion of the hybrid domain is a prerequisite for the unbending of the integrin ectodomain, which, in turn, is required for integrin activation according to current models. Neither did MD simulations of the ectodomain of α_5_β_1_ integrin bound to G*RGD*SP nor to taurocholic acid (TC) (Figure 1A) reveal such conformational changes, in line with results from immunofluorescence staining of IPRL that did not reveal an appearance of the active conformation of β_1_-integrin upon addition of TC either. The bile acids glycochenodeoxycholic acid (GCDC), taurochenodeoxycholic acid (TCDC), or taurolithocholic acid 3-sulfate (TLCS) were likewise ineffective with respect to β_1_ integrin activation according to immunofluorescence staining. All these bile acids differ from TUDC with respect to the configuration and/or presence or absence of functional groups in the cholane moiety.

In contrast, the taurine conjugate (T*nor*UDCA) of *nor*UDCA (Figure 1A), a C_23_ homolog of UDCA that lacks a methylene group in the sidechain, is moderately effective in exerting anticholestatic effects in experimental hepatocellular cholestatis [5]. Preliminary results from immunofluorescence staining of IPRL indicate that T*nor*UDCA and *nor*UDCA can activate β_1_ integrins, with stronger effects observed with *nor*UDCA. Another sidechain modification occurs if glycine rather than taurine is conjugated with the bile acid in the terminal synthesis step. Preliminary results from immunofluorescence staining indicate that the glyco-conjugated UDCA (GUDC; Figure 1A) does not activate β_1_ integrins although GUDC can be transported by the Ntcp [6]. In order to investigate the bile acids’ modes of action at a molecular level, we subjected *nor*UDCA and GUDC bound to the ectodomain of α_5_β_1_ integrin to MD simulations, employing the same setup as for the simulations above. In addition, we also performed MD simulations of the complex of α_5_β_1_ integrin with the unconjugated UDCA as well as of a ligand-free structure of the α_5_β_1_ ectodomain for reference. Together with the above results for TUDC and TC-bound α_5_β_1_ integrin, these simulations reveal a significant correlation between characteristic conformational changes in the βA domain and the potential of the bile acid to activate β_1_ integrins as observed in immunofluorescence staining: I) the higher this potential (TUDC, *nor*UDCA), the less is helix α_1_ kinked and the more is helix α_7_ tilted with respect to the ligand-free structure; II) changes in the opposite direction are observed for the non-activating bile acids (TC, GUDC); III) the MD simulations reveal that changes of helix α_1_ towards an activated integrin state are more pronounced than those of helix α_7_. According to these preliminary results, we predict that UDCA does not activate β_1_ integrins because the conformational characteristics of helices α_1_ and α_7_ observed with this bile acid do not differ much from those of the ligand-free structure. Finally, the MD simulations suggest that the cholane scaffolds of TUDC and *nor*UDCA adopt different binding modes in the cleft between the propeller and βA domains of α_5_β_1_ integrin; yet, the activating effects of both bile acids is funneled through helix α_1_ and from there leads to allosteric changes in the βA domain that propagate towards the hybrid domain.

In summary, in a combined computational and experimental study, we showed that TUDC directly activates α_5_β_1_ integrins inside hepatocytes and induces conformational changes in the β_1_ subunit that lead to integrin activation and swelling-independent signaling towards choleresis. A bile acid with a *nor*ursodeoxycholane scaffold (*nor*UDCA) was shown to activate β_1_ integrins even without conjugation. In contrast, bile acids modified in the cholane scaffold (TC, TCDC, TLCS) or conjugated to glycine (GCDC, GUDC) were shown to be non-activating. This suggests a unique role of the (*nor*)ursodeoxycholane scaffold for direct interaction with and activation of α_5_β_1_ integrins in connection with no or a taurine conjugation.
